# Evolutionary analysis of FAM83H in vertebrates

**DOI:** 10.1371/journal.pone.0180360

**Published:** 2017-07-06

**Authors:** Wushuang Huang, Mei Yang, Changning Wang, Yaling Song

**Affiliations:** The State Key Laboratory Breeding Base of Basic Science of Stomatology (Hubei-MOST) and Key Laboratory of Oral Biomedicine Ministry of Education, School and Hospital of Stomatology, Wuhan University, Wuhan, China; University of Naples Federico II, ITALY

## Abstract

Amelogenesis imperfecta is a group of disorders causing abnormalities in enamel formation in various phenotypes. Many mutations in the *FAM83H* gene have been identified to result in autosomal dominant hypocalcified amelogenesis imperfecta in different populations. However, the structure and function of FAM83H and its pathological mechanism have yet to be further explored. Evolutionary analysis is an alternative for revealing residues or motifs that are important for protein function. In the present study, we chose 50 vertebrate species in public databases representative of approximately 230 million years of evolution, including 1 amphibian, 2 fishes, 7 sauropsidas and 40 mammals, and we performed evolutionary analysis on the FAM83H protein. By sequence alignment, conserved residues and motifs were indicated, and the loss of important residues and motifs of five special species (Malayan pangolin, platypus, minke whale, nine-banded armadillo and aardvark) was discovered. A phylogenetic time tree showed the *FAM83H* divergent process. Positive selection sites in the C-terminus suggested that the C-terminus of *FAM83H* played certain adaptive roles during evolution. The results confirmed some important motifs reported in previous findings and identified some new highly conserved residues and motifs that need further investigation. The results suggest that the C-terminus of FAM83H contain key conserved regions critical to enamel formation and calcification.

## Introduction

Amelogenesis imperfecta (AI) is a clinically and genetically diverse group of human inherited disorders that exhibit enamel malformation of both primary and permanent dentitions with or without nondental phenotypes [1]. The enamel phenotype in AI can be broadly classified as hypoplastic, hypocalcified and hypomaturated. To date, mutations in 9 genes have been implicated in causing nonsyndromic AI in different phenotypes including *AMELX*, *ENAM*, *KLK4*, *MMP20*, *FAM83H*, *WDR72*, *C4ORF26*, *SLC24A4*, and *AMBN* [2–10]. Among them, as far as we know, *FAM83H* accounts for the most AI cases and is considered to be the main causative gene in autosomal dominant hypocalcified AI (ADHCAI).

Human *FAM83H* comprises five exons and encodes a non-secreted protein with 1179 amino acids (aa) in a region of 2.1 MB on chromosome 8 [6, 11]. The expression of FAM83H is ubiquitous; in addition to expression during tooth development [12], it can also be observed in the eyes, kidney, liver, bladder and larynx [6, 13]. To date, a total of nearly 23 mutations in *FAM83H* have been revealed to cause ADHCAI in different populations [13–15]. All identified mutations localize within the last exon of *FAM83H*, never before Ser^287^ or after Glu^694^ [13], and belong to truncated mutations except for a missense variant, which indicates the C-terminus of FAM83H, especially for the region after aa694, should be the key domain for enamel formation and mineralization. Researchers have been inclined to believe that a dominant negative effect rather than haploinsufficiency is the more likely molecular mechanism for *FAM83H* mutations resulting in ADHCAI [14, 16]. Previous studies have indicated that the N-terminus of FAM83H could contain a phospholipase D (PLD)-like domain and form dimers through this domain as PLD does [14]. Another study noted that the quite different predicted structures of the N-terminus and C-terminus of FAM83H imply they have different roles [17]. The latest research suggests that *FAM83H* mutations mediate the disorganization of the keratin cytoskeleton and then disrupt the desmosomes in ameloblasts [18]. Although many studies have focused on the molecular mechanism of FAM83H during enamel formation, the structure and function of FAM83H have yet to be further explored.

Evolutionary analysis is an alternative for revealing residues or motifs that are important for the structure and function of proteins and for predicting the association between amino acid alterations and genetic diseases [19]. It is based on the following premises: i) fundamental residues or motifs responsible for key functions of genes must be highly conserved; and ii) the selected sequences must cover a long period of evolution to ensure that the species are representative of different classes [20]. In the present study, we chose 50 vertebrate species representative of approximately 230 million years of evolution, including 1 amphibian, 2 fishes, 7 sauropsidas and 40 mammals, and performed evolutionary analysis on FAM83H to explore the sequence differences and the association between the sequence alterations and the tooth phenotypes among these species and to predict the essential residues and motifs of the protein function.

## Materials and methods

### Sequences & alignment

The coding sequences of *FAM83H* from 50 vertebrate species were selected from the NCBI and Ensembl databases (S1 Table). There were 5 species that have peculiar tooth development: i) the living Malayan pangolin lacks teeth as an adult, though vestigial teeth are found but resorbed before birth; ii) the minke whale is toothless; iii) the nine-banded armadillo teeth are covered with a thin layer of enamel as juveniles, but no prismatic layer of enamel can be identified in permanent teeth, and in both dentitions, the enamel layer is rapidly abraded and disappears; iv) the aardvark lacks enamel, and the front teeth are shed after birth and are not replaced; v) the platypus has teeth as a juvenile but loses them as an adult [21].

We aligned and analyzed the coding sequences of *FAM83H* from these 50 species using MEGA 7.0 (http://www.megasoftware.net/) [22]. Then, the protein sequences were aligned and divided into different groups to detect the conversed amino acids and motifs in FAM83H using the MUSCLE program in MEGA 7.0. In the present study, a motif is defined as a sequence motif by a short amino-acid sequence pattern that is conjectured to have some biological significance, and the site is defined as the specific amino acid. The groups were as follows: group 1, FAM83H protein sequences from all selected vertebrate species except for the 5 species listed above; group 2, FAM83H protein sequences from all selected mammal species except the 5 species listed above; group 3, FAM83H protein sequences from human and the 5 species listed above.

### Phylogenetic analysis

A phylogenetic time tree was constructed using the Reltime method in MEGA 7.0 [23]. First, we used the Clustal W program to align coding sequences of *FAM83H* in 40 mammals. Then, the maximum-likelihood (ML) method based on the GTR+G model (GTR: General Time Reversible; G: a discrete Gamma distribution) was used to construct an ML tree. The program Reltime was used to estimate divergence time. After providing a sequence alignment and a tree topology to the aforementioned mammals, we selected platypus as an outgroup. A single calibration constraint was used [24]; the minimum and maximum constraints were 92.1 and 116.8 million years (mya), respectively, for Placentalia divergence. The calibration constraints were inferred according to a previously proposed method [25]. In the dialogue of analysis preferences, we used the default settings.

### Selection tests

To explore the evolutionary process and to clarify the key sites in *FAM83H* during evolution, we performed a selection test based on the ratio of the nonsynonymous/synonymous substitution rate (ω = d_N_/d_S_) [26]. Values of ω>1, ω = 1, and ω<1 correspond to positive selection, neutral selection, and negative selection, respectively [26]. We used the Codeml program of the PAML 4.9 package to test the selective constraints working on *FAM83H* among mammals [27]. Based on the mammalian tree topology indicated by Meredith et al. [25], we drew a tree topology including 40 mammals manually using MEGA 7.0 for a tree file of PAML. We used the coding sequences of *FAM83H* of 40 mammals as sequence files running in PAML. Ambiguous sites and gaps of sequence alignment were included in our study. Site models for codon sequences were selected to test adaptive molecular evolution and detect amino acid sites under positive selection [26]. The selected models included M0, 1, 2, 3, 7, and 8. Two pairs of models, which form two likelihood ratio tests (LRT) of positive selection, seem to be especially useful. The first compares M1a (nearly neutral) with M2a (positive selection), and the second compares M7 (beta) with M8 (beta &ω) [26, 28]. When comparing the two models M1a (null) and M2a (alternative) as well as M7 (null) and M8 (alternative), the strength of support for the model M2a relative to M1a and for the model M8 relative to M7 is often assessed using the LRT [27]. A likelihood ratio test decides between a null model and alternative model by comparing the LRT to Χ^2^_2_. If the LRT >Χ^2^_2,_ the alternative model is selected, otherwise the null model is selected [27]. The Bayesian empirical Bayes (BEB) values estimated from M2a and M8 were used to identify sites under significant positive selection [28].

## Results

**Fig 1:**
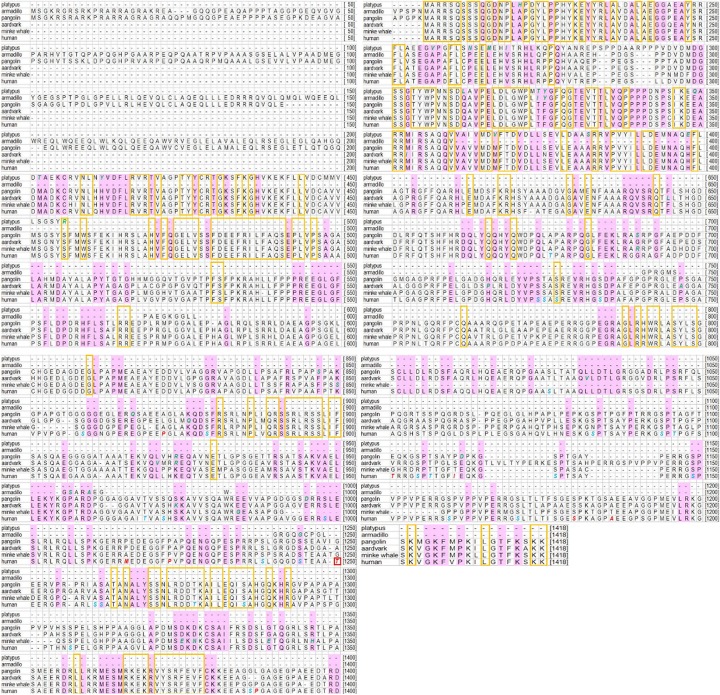
Alignment of FAM83H protein sequences among human and the five special species. Conserved amino acids in vertebrates excluding the five special species are indicated in the yellow box; conserved amino acids in mammals excluding the five special species are highlighted in the pink fill and in the yellow box; substitutions of conserved sites in the five special species are indicated in green, bold and italic font; phosphorylation sites in human FAM83H are presented in blue, bold and italic font; positive selection residues are shown in red, bold and italic font with a red border in the significant selection site.

The FAM83H protein sequences of 50 vertebrate species were aligned and analyzed. Human FAM83H was chosen as the reference sequence. In group 1, we examined the conserved residues of FAM83H in 45 vertebrate species excluding the 5 special species (platypus, Malayan pangolin, minke whale, nine-banded armadillo and aardvark). There are 179 conserved residues in FAM83H among the 45 vertebrate species (highlighted in the yellow box of Fig 1; the original data is in the S1 File): 85 sites are in exons 2–4 of human *FAM83H* (encoding aa1-246); 94 sites are in exon 5 of human *FAM83H* (encoding aa247-1179) and all phosphorylation sites are in exon 5. In group 2, there are up to 449 conserved residues of FAM83H in 35 mammals excluding the 5 special species (highlighted in the pink fill and in the yellow box of Fig 1; the original data is in the S2 File). Among them, we paid more attention to some specially conserved sites or motifs. In the N-terminal, human FAM83H aa162-170 (aa372-380 in Fig 1) and aa270-281 (aa480-491 in Fig 1) are conserved in vertebrates or mammals. The numbering in aa162-170 outside of the parentheses denotes the position of the amino acid in the published human FAM83H protein sequence (ENSP00000373565), while the numbering in aa372-380 within the parentheses denotes the position of the amino acid in human FAM83H in Fig 1, and the same is true for the following numbering. In the C-terminal after Glu^694^, there are some assembled conserved residues. Motifs such as K^733^VAELLEKY^741^ except E^736^ (K^946^VAELLEKY^954^ in Fig 1), R^784^SLESCLL^791^ except S^788^ (R^997^SLESCLL^1004^ in Fig 1), Q^818^LLDTLG^824^ (Q^1041^LLDTLG^1047^ in Fig 1) and S^1085^DKDKCSAI^1093^ except K^1089^ (S^1324^DKDKCSAI^1332^ in Fig 1) are conserved in mammalian species. The region between aa1025-1055 (aa1262-1292 in Fig 1) contains 23 conserved residues including 18 sites conserved in vertebrates, and S^1025^ (S^1262^ in Fig 1), T^1040^ (T^1277^ in Fig 1), and S^1048^ (S^1285^ in Fig 1) are phosphorylation sites. AA1123-1141 (aa1362-1380 in Fig 1) are conserved and contain 12 amino acids conserved in vertebrates. In region aa1160-1179 (aa1399-1418 in Fig 1), there are 12 sites conserved in mammals including 4 sites conserved in vertebrates. There are also some sporadically conserved sites in the C-terminus of FAM83H.

In group 3, for FAM83H protein sequences, there are 251 aa in platypus, 1377 aa in Malayan pangolin, 1104 aa in minke whale, 384 aa in nine-banded armadillo and 1197 aa in aardvark. The alignment of sequences of the five special species with human FAM83H protein sequences is shown in Fig 1. Platypus and nine-banded armadillo FAM83H severely lost amino acids in the C-terminus. Malayan pangolin and nine-banded armadillo FAM83H have 200 more amino acids than human in FAM83H in the N-terminus. From Fig 1, we can see that the 179 conserved sites in the 45 vertebrates are also conserved among human and the 5 special species except for the deletion sequences in platypus and nine-banded armadillo and one site S^246^ (S^456^ in Fig 1) in human FAM83H, which is substituted by R in platypus. Except for the N-terminus variation of pangolin FAM83H, the difference in conserved residues between Malayan pangolin and human FAM83H is small. Human E^899^ (E^1112^ in Fig 1), E^782^ (E^995^ in Fig 1), K^711^ (K^924^ in Fig 1), E^655^ (E^867^ in Fig 1), F^636^ (F^847^ in Fig 1), and G^479^ (G^690^ in Fig 1) are conversed residues in mammalian FAM83H but are substituted by amino acids D, D, R, Q, S, and S, respectively, in pangolin. The Minke whale lacks 59 continuous residues which correspond to those of human FAM83H aa779-837 (aa992-1050 in Fig 1) and has some other deletions and substitutions in conserved sites. The aardvark FAM83H protein sequence shows a high similarity to that of human, except for several substitutions that occur only in conserved sites of mammals. For example, human M^143^ (M^353^ in Fig 1), F^434^ (F^645^ in Fig 1), H^505^ (H^716^ in Fig 1), P^536^ (P^747^ in Fig 1), K^664^ (K^876^ in Fig 1), L^708^ (L^921^ in Fig 1), K^711^ (K^924^ in Fig 1), S^761^ (S^974^ in Fig 1), and L^819^ (L^1032^ in Fig 1) are conversed residues in mammalian FAM83H but are substituted by amino acids I, L, P, A, Q, V, R, K, and V, respectively, in aardvark.

**Fig 2:**
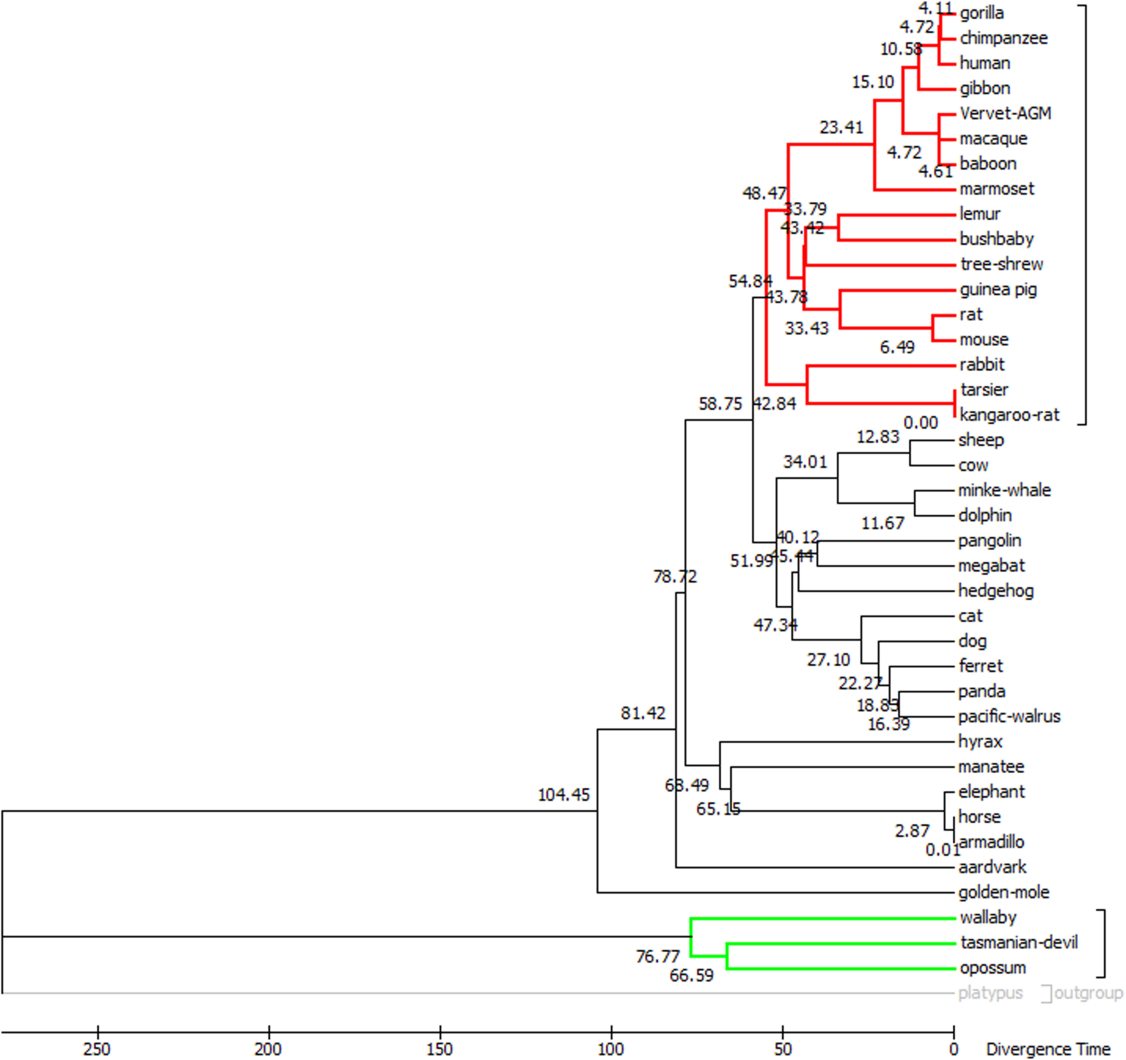
The phylogenetic time tree for *FAM83H* in 40 mammals. The time tree was computed using the Reltime method (GTR+G model) involving 40 mammalian *FAM83H* coding sequences under one calibration constraint. The platypus was selected as an outgroup. There are a total of 6441 positions with gaps in the final dataset. The phylogenic and divergence timescales were implemented in MEGA 7.0. Euarchontoglires (red) and marsupialia (green) are highlighted.

In the present study, a different divergent process of *FAM83H* among Mammalia is indicated in Fig 2. For example, *FAM83H* diverged 54.84 mya in Euarchontoglires and diverged 76.77 mya in Marsupialia. For the five special species, *FAM83H* diverged 81.42 mya in aardvark, 40.12 mya in Malayan pangolin, 11.67 mya in minke whale and 0.01 mya in nine-banded armadillo. For platypus, the divergence time is not presented because the Reltime method did not apply to this outgroup.

The results of the selection test are shown in Table 1. The LRT statistic for comparing models M1a and M2a was much smaller than the critical values from theΧ_2_^2^. However, the test using models M7 and M8 presented a different result. The M8 model was significantly favored because the LRT statistic for comparing models M7 and M8 was much greater than the critical values from theΧ_2_^2^. This meant that there were some sites in FAM83H subjected to positive selection. The empirical Bayes approach was implemented to find codon sites under positive selection. A total of 19 positive selection sites were identified. Excluding the existing gaps and ambiguous sites, we rearranged the rest of the 9 sites for matching the accurate positions of the human FAM83H protein. The positive selection sites 945, 1246, 1256, 1377, 1383, 1634, 1646, 1699, and 1847 correspond to human FAM83H P^660^ (P^872^ in Fig 1), T^888^ (T^1101^ in Fig 1), I^898^ (I^1111^ in Fig 1), S^945^ (S^1180^ in Fig 1), A^951^ (A^1186^ in Fig 1), M^981^ (M^1216^ in Fig 1), P^988^ (P^1223^ in Fig 1), T^1014^ (T^1250^ in Fig 1), and P^1148^ (P^1387^ in Fig 1), respectively (all 9 sites are shown in red, bold and italic font in Fig 1). All these sites were on the C-terminus of FAM83H. Only one site T^1014^ was under significant positive selection withω = 1.48±0.12 and posterior probability (pp) >95% (shown in red, bold and italic font with red border in Fig 1). We also performed selection test using SLAC method in HyPhy (http://hyphy.org/). The procedures of the SLAC method, the results of selection test (shown in S1 Fig) were described in detail in S3 File. SLAC analysis identified three positively selected sites, and only one site was significant. They are corresponding to human FAM83H L^709^ (*P* = 0.0962), T^888^ (*P* = 0.0671), S^1177^ (*P* = 0.0248).

**Table 1:** The results of selection tests.

Site model	Likelihood scores		LRT		Positively selected sites
			-2ΔL (df)	*P*-value	
40 mammals	M1a =	M2a =	0	-	1246 1634 1646 1699 1847
	-51068.04	-51068.04			
	M7 =	M8 =	96.50(2)	*P* <0.001	**183(ω = 1.23±0.35)**
					**185(ω = 1.63±0.00)**
	-50703.33	-50655.08			**187(ω = 1.37±0.00)**
					194(ω = 4.67±0.00)**
					**582(ω = 1.76±0.00)**
					**761(ω = 1.48±0.00)**
					945(ω = 1.30±0.32)
					**1129(ω = 2.40±0.00)**
					1246(ω = 1.40±0.24)
					1256(ω = 1.22±0.35)
					**1341(ω = 1.49±0.00)**
					1377(ω = 1.15±0.36)
					1383(ω = 1.30±0.32)
					**1386(ω = 1.73±0.00)**
					1634(ω = 1.42±0.22)
					1646(ω = 1.41±0.23)
					1699(ω = 1.48±0.12)*
					**1841(ω = 1.56±0.00)**
					1847(ω = 1.38±0.26)

LRT: likelihood ratio tests

Positions that occurred in ambiguous sites are bold

Posterior probability (pp)

** *P* > 99%

* *P* > 95%

## Discussion

Previous studies proposed that FAM83H could interact with its wild-type or mutant protein or other proteins to play a role in intracellular molecular transportation, regulation of cytoskeletal networks, and enamel formation [11, 14, 17, 29]. Highly conserved residues or motifs among species indicate key functions of proteins. In the present study, we identified many conserved residues and motifs in the FAM83H protein. In the N-terminus, human FAM83H aa162-170 (aa372-380 in Fig 1) is conservative in mammals, for which a previous study predicted aa162-170 as a motif that is common to the transactivation domains [6]. The present study showed human FAM83H aa270-281 (F^270^DEEFRILFAQS^281^) (aa480-491 in Fig 1) is conserved in vertebrates. Previous research identified that F-X-X-X-F was a CK1-binding site, and that wild type FAM83H, mutant FAM83H^1-287^, as well as mutant FAM83H^1-697^ can interact with CK1 [14, 29]. All these suggest that F^270^DEEFRILF^278^ play a significant role in FAM83H function. Human FAM83H exon 5 encoding aa247-1179 is relatively variable compared to exons 2–4, but all phosphorylation sites are located in exon 5 and there are some conserved motifs after Glu^694^. Protein sequence alignment showed that K^733^VAELLEKY^741^ except for E^736^ (K^946^VAELLEKY^954^ in Fig 1), R^784^SLESCLL^791^ except for S^788^(R^997^SLESCLL^1004^ in Fig 1), Q^818^LLDTLG^824^ (Q^1041^LLDTLG^1047^ in Fig 1), S^1085^DKDKCSAI^1093^ except for K^1089^ (S^1324^DKDKCSAI^1332^ in Fig 1), aa1025-1055 (aa1262-1292 in Fig 1) and aa1123-1141 (aa1362-1380 in Fig 1) are conserved during evolution. 22 AI-causing *FAM83H* mutations in human would truncate the protein, never before Ser^287^ or after Glu^694^ [13, 14], which indicates that conserved residues and motifs after Glu^694^ have an essential influence on enamel formation. Only one missense mutation was identified in the C-terminus of *FAM83H* and was not a conserved site [15]. All truncated mutations in *FAM83H* would definitely result in the loss of the C-terminus and amounts of conserved amino acids. Moreover, the loss of the C-terminus of FAM83H would cause a reduction in phosphorylation sites and change its three-dimensional structure. Previous studies demonstrated that the N-terminus of FAM83H interacted with CK1 and the C-terminus interacted with keratins, and that truncated mutant FAM83H could interact with CK1, but it would lose normal interaction with keratins and then result in a disassembled keratin cytoskeleton with the disappearance of keratin filaments and desmosomes [14, 18, 29]. Thus, it can be inferred that mutant FAM83H disrupts the formation of desmosomes among pre-ameloblasts and cell-cell interactions and consequently disturbs the formation or the function of ameloblasts in the secretory stage, finally leading to ADHCAI. To date, the identified *FAM83H* mutations have not been reported to result in other disorders in human except for ADHCAI. In other species, only one study reported a nonsense mutation in canine *FAM83H* causing CKCSID (congenital keratoconjunctivitis sicca and ichthyosiform dermatosis) [30]. Because both ADHCAI and CKCSID are related to epithelial disorders, it is proposed that FAM83H could be involved in the differentiation and function of epithelial cells.

In the present study, all identified positive selection sites are on the C-terminus of FAM83H, according to the results of Codeml program in PAML and the results of SLAC method in HyPhy. The positive selection sites usually indicated that the substitution of amino acids in these sites could be adaptive or advantageous during the evolutionary process. Though only one site is significant, both in Codeml method and in SLAC program, the substitutions of the rest of the positive sites could also be advantageous during evolution, and this probability cannot be ruled out. Only some sites in FAM83H are under positive selection, which means the rest of the sites would be either under neutral selection or under negative selection, and according to the results of the sequence alignments, there are many conserved sites in FAM83H (179 conserved residues in vertebrates and 449 conserved residues in mammals). Taking the above points into account, we inferred that the gene *FAM83H* is evolutionarily conserved in most sites and is functionally constrained. On the one hand, many conserved sites or motifs and all identified ADHCAI-causing mutations were in the C-terminus of FAM83H, which indicated that the C-terminus of *FAM83H* could be critical to essential gene function and enamel formation. On the other hand, the inferred positive selection sites assembling in the C-terminus suggested that the C-terminus of *FAM83H* also played some adaptive roles during evolution.

The speciation time of the mammalia remains controversial in phylogenetics, especially during the Cretaceous and the Cretaceous-Paleogene [25, 31]. In this study, a chronogram of *FAM83H* was constructed by applying the Reltime method in MEGA 7.0. The time tree inferred in this study presents some differences from that of previous studies [31, 32]. The variety of species, quantity of mammals and research techniques could have contributed to the differences. Moreover, variation in selection pressure on different genes also may have affected the timing of divergence. More attention was paid to the *FAM83H* divergence time of the five special species, and the time of molecular analysis and fossil records was compared. Pangolin appeared in the middle Eocene (40 Ma) according to the fossil record [33], and the root of aardvark can be traced back to the late Cretaceous or late Paleocene (75–65 Ma) [34]. The fossil records are similar in terms of the divergence time of *FAM83H* in these two species in our study (Malayan pangolin: 40.12 Ma; aardvark: 81.42 Ma), which indicates that *FAM83H* diverged during the origins of these two species, not during the evolutionary processes. Existing baleen whales are toothless but their ancestors had teeth [21]. Minke whale belongs to the edentulous mysticetes whose most ancient family lived from the late Oligocene to the Pliocene (25–5 Ma) [21], whereas minke whale *FAM83H* diverged approximately 11.67 Ma ago. Thus, we could not infer that minke whale tooth loss was earlier or *FAM83H* diverged earlier according to our study. Armadillo diverged from its ancestors during the Eocene (55–33 Ma) [21]. In our study, the nine-banded armadillo *FAM83H* diverged late (0.01 Ma), suggesting that this gene evolved during the evolutionary process of the armadillo and not in its speciation time. Moreover, the nine-banded armadillo is the only xenarthran species ranging in North America, while all the other living xenarthrans are restricted to Central and South America [21]. These regional differences may have contributed to *FAM83H* divergence.

Knowing that *FAM83H* truncated mutations cause ADHCAI in human, we wondered about the difference in protein sequences between tooth-less or enamel-less species and human. We selected 3 tooth-less species (platypus, Malayan pangolin and minke whale) and 2 enamel-less species (nine-banded armadillo and aardvark) and compared their FAM83H protein sequences with that of human. These species all have a pre-adapted “tool” to help in food uptake or processing to compensate for their lack of teeth or enamel, such as a beak in the platypus, an elongated sticky tongue in the Malayan pangolin, baleen in the minke whale and hypsodonty/hypselodonty in the nine-banded armadillo and aardvark [21]. It is not clear whether the FAM83H sequence feature in these species is relevant to selective pressures associated with the following aspects: i) the quantity of enamel in the embryonic tooth structures of the pangolin, minke whale and platypus; ii) the FAM83H expression during the development of temporary teeth regulating the responses of nearby tissues, such as a beak or elongated tongue; and iii) the importance of expression of FAM83H in other tissues. In the alignment, platypus and nine-banded armadillo FAM83H severely lost amino acids in the C-terminus. Platypus lack teeth as adults while nine-banded armadillo lack enamel. The enamel layer in nine-banded armadillo teeth is easily worn and then disappears [21], which is similar to the phenotype of human AI resulting from *FAM83H* truncated mutations. Adult platypus develop keratinized pads instead of the cusped molars in juvenile platypus [21]. All these indicate that the C-terminal region of FAM83H could be essential for enamel calcification and responsible for oral epithelial formation. However, it is important to note that other enamel-related proteins are inactivated in some of these species, such as AMBN and ENAM pseudogenization in mysticeti [35]; ENAM pseudogenization in aardvark, pangolin, cetacea and armadillo [36]; AMTN pseudogenization in armadillo [19]; KLK4 missing in the genomes of aardvark and nine-banded armadillo and deleterious mutations found in KLK4 in minke whale [37]; AMBN pseudogenization in aardvark, and either inactivated or encoded defective proteins in armadillo [38]. It is not certain that the lack of teeth or enamel results from important residue loss of FAM83H or other genes being inactivated or the joint action of both. Molecular mechanisms of FAM83H in amelogenesis have yet to be further explored.

Evolutionary analysis implemented on vertebrate FAM83H sequences helped us in learning more about FAM83H: (i) the identified highly conserved residues or motifs among species further indicated their importance and key role in the function of FAM83H; (ii) the *FAM83H* divergence time provided a clue for its evolutionary process; (iii) positive selection sites in the C-terminus suggested that the C-terminus of *FAM83H* played certain adaptive roles during evolution; and (iv) the C-terminus of FAM83H contained key conserved regions critical to enamel calcification and epithelial formation based on analysis of special species. Further studies need to explore FAM83H structure and function in epithelial formation, its interaction with other proteins, and the pathogenic mechanism in amelogenesis imperfecta.

## Supporting information

S1 Table The selected species and FAM83H sequences in this study. (DOCX)

S1 File Alignment of FAM83H protein sequences among 45 vertebrates (excluding the five special species). Conserved residues are highlighted in yellow.
(XLS)

S2 File Alignment of FAM83H protein sequences among 35 mammalian species (excluding the five special species). Conserved residues are highlighted in yellow.
(XLS)

S3 File The selection test by SLAC in HyPhy. (DOCX)

S1 Fig The selection test results of SLAC in this study. (TIF)
