# Supplementary material for: Evolutionary analysis of FAM83H in vertebrates
Source: PLoS One. 2017 Jul 6;12(7):e0180360. doi: 10.1371/journal.pone.0180360 (PMC5500323; doi:10.1371/journal.pone.0180360)
Supplement: S1 Table — (DOCX) [file pone.0180360.s001.docx]

**S1 Table. The selected species and FAM83H sequences in this study.**

| Species | Sequence ID | Database |
| --- | --- | --- |
| Human(homo sapiens) | ENST00000388913 | Ensembl |
| Chimpanzee(Pan troglodytes) | ENSPTRT00000059775 | Ensembl |
| Gorilla(gorilla gorilla) | ENSGGOT00000007944 | Ensembl |
| Northern white-cheeked gibbon(Nomascus leucogenys) | XM_012507180 | NCBI |
| Vervet-AGM(Chlorocebus sabaeus) | ENSCSAT00000006977 | Ensembl |
| Macaque(Macaca mulatta) | ENSMMUT00000020003 | Ensembl |
| Olive baboon(Papio anubis) | ENSPANT00000004609 | Ensembl |
| Marmoset(Callithrix jacchus) | [ENSCJAT00000042272](http://asia.ensembl.org/Callithrix_jacchus/Transcript/Summary?db=core;g=ENSCJAG00000021492;r=16:50936493-50941450;t=ENSCJAT00000042272) | Ensembl |
| Bushbaby(Otolemur garnettii) | [ENSOGAT00000027713](http://asia.ensembl.org/Otolemur_garnettii/Transcript/Sequence_cDNA?db=core;g=ENSOGAG00000025465;r=GL873764.1:1161028-1165537;t=ENSOGAT00000027713) | Ensembl |
| Philippine tarsier(Tarsius syrichta) | XM_008056730 | NCBI |
| grey Mouse Lemur(Microcebus murinus) | XM_012760130 | NCBI |
| Mouse(Mus musculus) | ENSMUST00000170153 | Ensembl |
| Rat(Rattus norvegicus) | ENSRNOT00000082433 | Ensembl |
| Ord’s kangaroo rat(Dipodomys ordii) | XM_013033836 | NCBI |
| Guinea Pig(Cavia porcellus) | ENSCPOT00000013453 | Ensembl |
| Rabbit(Oryctolagus cuniculus) | XM_008253707 | NCBI |
| Tree Shrew(Tupaia belangeri) | [ENSTBET00000006518](http://asia.ensembl.org/Tupaia_belangeri/Transcript/Summary?db=core;g=ENSTBEG00000006501;r=GeneScaffold_4879:3-5509;t=ENSTBET00000006518) | Ensembl |
| Cow(Bos taurus) | [ENSBTAT00000019225](http://asia.ensembl.org/Bos_taurus/Transcript/Summary?db=core;g=ENSBTAG00000038682;r=14:2227350-2232020;t=ENSBTAT00000019225) | Ensembl |
| Sheep(Ovis aries) | [ENSOART00000017899](http://asia.ensembl.org/Ovis_aries/Transcript/Summary?db=core;g=ENSOARG00000016436;r=9:13967503-13978556;t=ENSOART00000017899) | Ensembl |
| Minke whale(Balaenoptera acutorostrata scammoni) | XM_007166933 | NCBI |
| Dolphin(Tursiops truncatus) | [ENSTTRT00000015190](http://asia.ensembl.org/Tursiops_truncatus/Transcript/Summary?db=core;g=ENSTTRG00000015190;r=GeneScaffold_63:193972-198975;t=ENSTTRT00000015190) | Ensembl |
| Horse(Equus caballus) | XM_014728167 | NCBI |
| Pacific walrus(Odobenus rosmarus divergens) | XM_004400760 | NCBI |
| Cat(Felis catus) | [ENSFCAT00000029161](http://asia.ensembl.org/Felis_catus/Transcript/Summary?db=core;g=ENSFCAG00000025159;r=F2:81796027-81800751;t=ENSFCAT00000029161) | Ensembl |
| Dog(Canis lupus familiaris) | [ENSCAFT00000049368](http://asia.ensembl.org/Canis_familiaris/Transcript/Summary?db=core;g=ENSCAFG00000032391;r=13:37323386-37330180;t=ENSCAFT00000049368) | Ensembl |
| Panda(Ailuropoda melanoleuca) | [ENSAMET00000007105](http://asia.ensembl.org/Ailuropoda_melanoleuca/Transcript/Summary?db=core;g=ENSAMEG00000006482;r=GL192891.1:987737-992251;t=ENSAMET00000007105) | Ensembl |
| Ferret(Mustela putorius furo) | [ENSMPUT00000008237](http://asia.ensembl.org/Mustela_putorius_furo/Transcript/Summary?db=core;g=ENSMPUG00000008168;r=GL897203.1:922297-926966;t=ENSMPUT00000008237) | Ensembl |
| malayan pangolin(Manis javanica) | XM_017646835 | NCBI |
| Megabat(Pteropus vampyrus) | [ENSPVAT00000005712](http://asia.ensembl.org/Pteropus_vampyrus/Transcript/Summary?db=core;g=ENSPVAG00000005711;r=scaffold_14070:28313-32816;t=ENSPVAT00000005712) | Ensembl |
| Hedgehog(Erinaceus europaeus) | [ENSEEUT00000005203](http://asia.ensembl.org/Erinaceus_europaeus/Transcript/Summary?db=core;g=ENSEEUG00000005198;r=GeneScaffold_7622:9370-19584;t=ENSEEUT00000005203) | Ensembl |
| Cape golden mole (Chrysochloris asiatica) | XM_006830746 | NCBI |
| Florida manatee(Trichechus manatus latirostris) | XM_004387380 | NCBI |
| Elephant (Loxodonta africana) | [ENSLAFT00000036487](http://asia.ensembl.org/Loxodonta_africana/Transcript/Summary?db=core;g=ENSLAFG00000029225;r=scaffold_146:556905-561601;t=ENSLAFT00000036487) | Ensembl |
| Hyrax(Procavia capensis) | [ENSPCAT00000006990](http://asia.ensembl.org/Procavia_capensis/Transcript/Summary?db=core;g=ENSPCAG00000007010;r=GeneScaffold_6470:11304-15347;t=ENSPCAT00000006990) | Ensembl |
| Aardvark(Orycteropus afer afer) | XM_007956087 | NCBI |
| Nine-banded armadillo (Dasypus novemcinctus) | XM_004454793 | NCBI |
| Tasmanian devil(Sarcophilus harrisii) | [ENSSHAT00000003803](http://asia.ensembl.org/Sarcophilus_harrisii/Transcript/Summary?db=core;g=ENSSHAG00000003316;r=GL841405.1:59368-92245;t=ENSSHAT00000003803) | Ensembl |
| Wallaby(Macropus eugenii) | [ENSMEUT00000003945](http://asia.ensembl.org/Macropus_eugenii/Transcript/Summary?db=core;g=ENSMEUG00000003926;r=GeneScaffold_8637:1618-15225;t=ENSMEUT00000003945) | Ensembl |
| gray short-tailed opossum (Monodelphis domestica) | XM_007488680 | NCBI |
| Platypus(Ornithorhynchus anatinus) | [ENSOANT00000000665](http://asia.ensembl.org/Ornithorhynchus_anatinus/Transcript/Summary?db=core;g=ENSOANG00000000413;r=Ultra734:53936-55287;t=ENSOANT00000000665) | Ensembl |
| Chinese softshell turtle(Pelodiscus sinensis) | [ENSPSIT00000003260](http://asia.ensembl.org/Pelodiscus_sinensis/Transcript/Summary?db=core;g=ENSPSIG00000003110;r=JH210032.1:95091-102966;t=ENSPSIT00000003260) | Ensembl |
| Zebra Finch(Taeniopygia guttata) | XM_012572152 | NCBI |
| Flycatcher(Ficedula albicollis) | [ENSFALT00000009080](http://asia.ensembl.org/Ficedula_albicollis/Transcript/Summary?db=core;g=ENSFALG00000008669;r=JH603220.1:6504440-6532934;t=ENSFALT00000009080) | Ensembl |
| Duck(Anas platyrhynchos) | [ENSAPLT00000010907](http://asia.ensembl.org/Anas_platyrhynchos/Transcript/Summary?db=core;g=ENSAPLG00000010481;r=KB743897.1:324760-331896;t=ENSAPLT00000010907) | Ensembl |
| Chicken(Gallus gallus) | [ENSGALT00000047899](http://asia.ensembl.org/Gallus_gallus/Transcript/Summary?db=core;g=ENSGALG00000033118;r=2:149292424-149298897;t=ENSGALT00000047899) | Ensembl |
| Turkey(Meleagris gallopavo) | [ENSMGAT00000014701](http://asia.ensembl.org/Meleagris_gallopavo/Transcript/Summary?db=core;g=ENSMGAG00000013067;r=GL429399.1:13583-18788;t=ENSMGAT00000014701) | Ensembl |
| green Anole (Anolis carolinensis) | XM_008123510 | NCBI |
| Xenopus(Xenopus tropicalis) | [ENSXETT00000011553](http://asia.ensembl.org/Xenopus_tropicalis/Transcript/Summary?db=core;g=ENSXETG00000005283;r=GL174256.1:8264-25700;t=ENSXETT00000011553) | Ensembl |
| Coelacanth(Latimeria chalumnae) | XM_006004009 | NCBI |
| Zebra fish(Danio rerio) | XM_005159768 | NCBI |
