# Supplementary material for: Evolutionary analysis of FAM83H in vertebrates
Source: PLoS One. 2017 Jul 6;12(7):e0180360. doi: 10.1371/journal.pone.0180360 (PMC5500323; doi:10.1371/journal.pone.0180360)
Supplement: S3 File — (DOCX) [file pone.0180360.s004.docx]

**Selection Test by SLAC Method**

**Method and Material**

The selection pressures acting on each site of 40 mammalian FAM83H coding sequences are tested with the codon-based SLAC method (Single Likelihood Ancestor Counting) in HyPhy (http://hyphy.org/) [[1](#_ENREF_1)]. 40 mammalian FAM83H coding sequences with ambiguous sites and gaps are specified as a codon data file. Based on the mammalian tree topology indicating by Meredith et al [[2](#_ENREF_2)], the tree topology is drawn manually by MEGA 7.0 for tree file. Enter a 6 character nucleotide model *012345*, *Estimate dN/dS only* option for the estimation of dN/dS, *Single likelihood ancestor* for the ‘Ancestor options’, selection analysis over the *Full tree*, *Averaged* for the treatment of ambiguities, *Approximate* for the test statistic, specify *p*-value of 0.05 as significant level for a site to be classified as positively selected.

**Results**

The analysis identified one positively selected sites 1876 corresponding to human FAM83H S^1177^ (S^1418^ in fig.1), with p-value *P*=0.0248. Other two sites, though *P*>0.05, the probability that they were under positive selection could not be ruled out: site 1002 corresponding to human FAM83H L^709^ (L^922^ in fig.1), with p-value *P*=0.0962; site 1246 corresponding to human FAM83H T^888^ (T^1101^ in fig.1), with p-value *P*=0.0671.


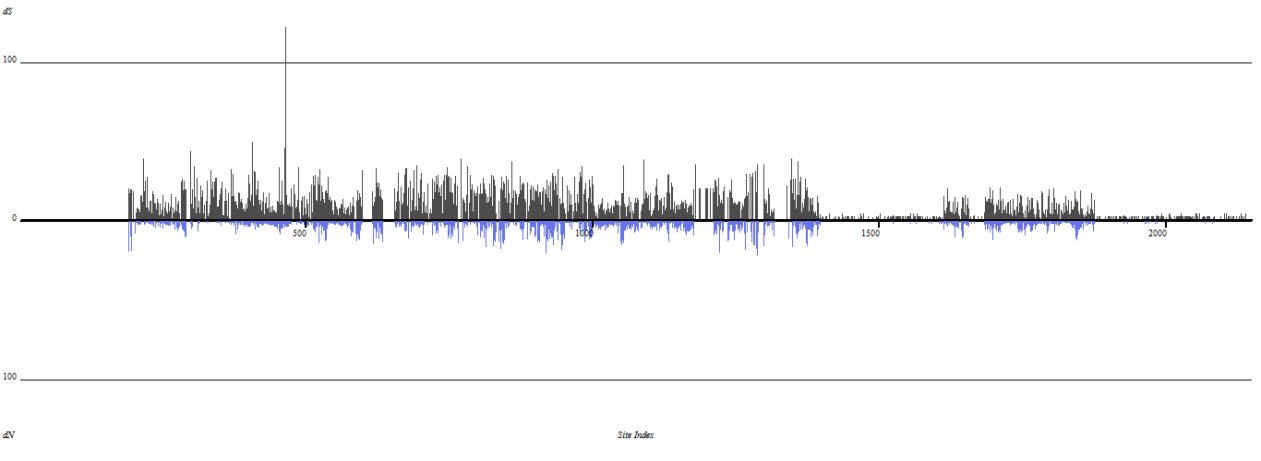


**Figure: The results of selection test in SLAC method.**

*dS* and *dN* estimated from the 40 mammalian sequences of FAM83H using SLAC.

**References**

1. Pond SL, Frost SD, Muse SV. HyPhy: hypothesis testing using phylogenies. Bioinformatics. 2005;21(5):676-9. doi: 10.1093/bioinformatics/bti079. PubMed PMID: 15509596.

2. Meredith RW, Janecka JE, Gatesy J, Ryder OA, Fisher CA, Teeling EC, et al. Impacts of the Cretaceous Terrestrial Revolution and KPg extinction on mammal diversification. Science. 2011;334(6055):521-4. doi: 10.1126/science.1211028. PubMed PMID: 21940861.
