# Supplementary figures and images for: Evolutionary analysis of FAM83H in vertebrates
Source: PLoS One. 2017 Jul 6;12(7):e0180360. doi: 10.1371/journal.pone.0180360 (PMC5500323; doi:10.1371/journal.pone.0180360)

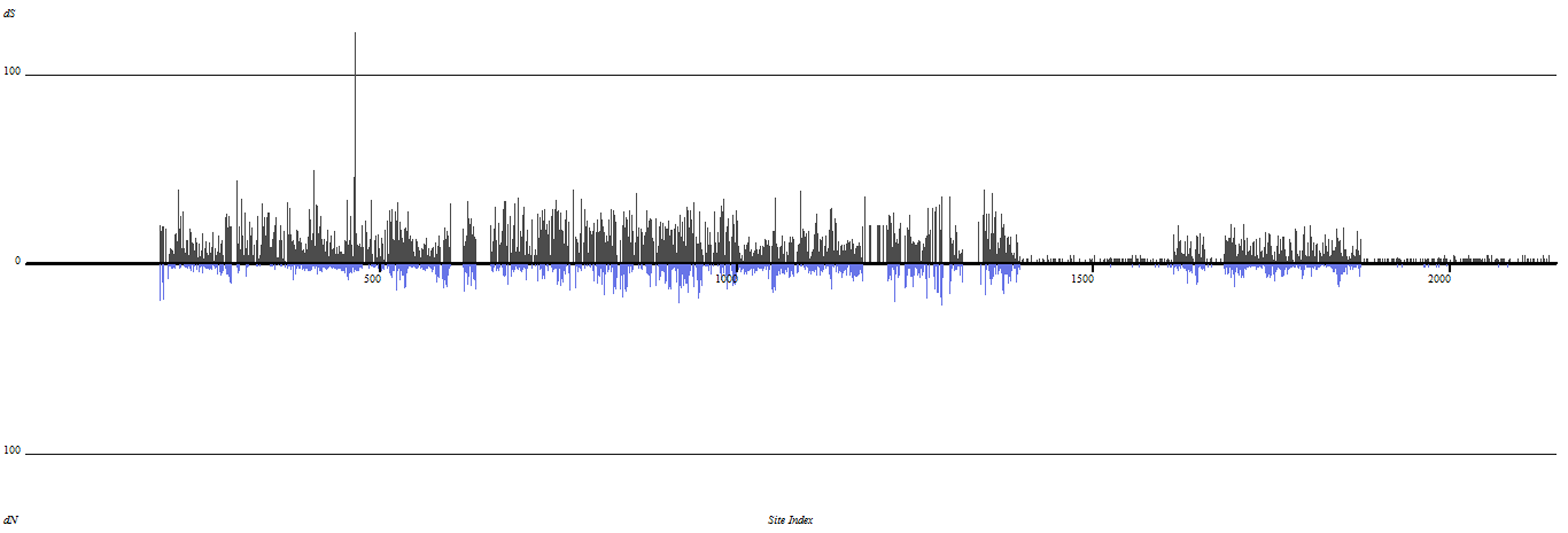

Supplement: S1 Fig — (TIF) [file pone.0180360.s005.tif]
